# Extending Inferential Group Analysis in Type 2 Diabetic Patients with Multivariate GLM Implemented in SPM8

**DOI:** 10.2174/1874440001711010032

**Published:** 2017-05-29

**Authors:** Fábio S. Ferreira, João M.S. Pereira, João V. Duarte, Miguel Castelo-Branco

**Affiliations:** 1 Institute for Biomedical Imaging and Life Sciences (IBILI), Faculty of Medicine, University of Coimbra, Portugal; 2 Laboratory of Biostatistics and Medical Informatics, Faculty of Medicine, University of Coimbra, Portugal; 3 Institute for Nuclear Sciences Applied to Health (ICNAS), University of Coimbra, Portugal

**Keywords:** SPM, VBM, T1, T2, Multivariate GLM, Type 2 diabetes mellitus

## Abstract

**Background:**

Although voxel based morphometry studies are still the standard for analyzing brain structure, their dependence on massive univariate inferential methods is a limiting factor. A better understanding of brain pathologies can be achieved by applying inferential multivariate methods, which allow the study of multiple dependent variables, *e.g.* different imaging modalities of the same subject.

**Objective:**

Given the widespread use of SPM software in the brain imaging community, the main aim of this work is the implementation of massive multivariate inferential analysis as a toolbox in this software package. applied to the use of T1 and T2 structural data from diabetic patients and controls. This implementation was compared with the traditional ANCOVA in SPM and a similar multivariate GLM toolbox (MRM).

**Method:**

We implemented the new toolbox and tested it by investigating brain alterations on a cohort of twenty-eight type 2 diabetes patients and twenty-six matched healthy controls, using information from both T1 and T2 weighted structural MRI scans, both separately – using standard univariate VBM - and simultaneously, with multivariate analyses.

**Results:**

Univariate VBM replicated predominantly bilateral changes in basal ganglia and insular regions in type 2 diabetes patients. On the other hand, multivariate analyses replicated key findings of univariate results, while also revealing the thalami as additional foci of pathology.

**Conclusion:**

While the presented algorithm must be further optimized, the proposed toolbox is the first implementation of multivariate statistics in SPM8 as a user-friendly toolbox, which shows great potential and is ready to be validated in other clinical cohorts and modalities.

## 1 INTRODUCTION

The understanding of the brain and related pathologies is hardly tackled when using a single neuroimaging modality, such as T1 magnet resonance (MR) imaging in volumetric studies or positron emission tomography (PET) in metabolic and neurochemical studies. The information that each imaging approach can provide is likely to be complementary [1]. As such, it is desirable to combine imaging methods in order to better ascertain the underlying mechanisms under analysis.

In particular, the popular approach voxel based morphometry (VBM) detects brain regions that exhibit some variation in brain tissue content, either correlated with a covariate or contrasting between cohorts [2]. Notably, the underlying statistics rely on using particular cases of the univariate General Linear Model (GLM), which lies at the basis of the statistical parametric maps yielded by testing hypothesis on regionally specific effects in neuroimaging data [3].

Although these univariate methods have been fundamental tools in modern neuroimaging, it is accepted that the presence of multivariate relationships between different brain regions, coupled with information provided by distinct imaging modalities in any single region, might not be explained by univariate analyses alone [2, 4, 5]. The wider application of multivariate statistical methods in brain morphometry is a definite need.

Focusing solely on inferential voxel-wise analyses rather than pattern recognition, this study presents a mass multivariate GLM method that is a natural extension of the mass univariate GLM approach used in VBM studies. This multivariate GLM can address multimodal group-level analyses using multiple MRI sequences. It should be noted that implementations of multivariate GLM solutions have already been applied in the context of neuroimaging in the previous studies [6, 7]. In the former study, the authors implemented the multivariate GLM in AFNI program 3dMVM in the open source statistical language R (R Core Team, 2013) to handle inherent problems of univariate GLM in the presence of multiple within-factors, or when quantitative covariates are involved in the presence of a within-subject factor. In the latter study, the authors implemented a MATLAB-based toolbox to model both repeated-measures and multimodal group-level imaging data. Other implementations can be found, *e.g.* in FSL (http://fsl.fmrib.ox.ac.uk/fsl/fslwiki/PALM). The authors, however, are not aware of any such solution implemented within the SPM software, which is one of the most popular toolboxes used in the context of neuroimaging and likely the most popular tool for VBM studies. This works presents for the first time an easy-to-use implementation of multivariate GLM analyses directly in SPM (version 8 - http://www.fil.ion.ucl.ac.uk/spm/software/spm8).

In order to demonstrate the potential of adapting the SPM software for mass multivariate analyses, this study uses, within the same model, gray matter volumetric information extracted from T1- and T2-weighted structural MRI scans of type 2 diabetes mellitus (T2DM) patients. T1 images have been widely used in VBM studies to assess gray matter changes, while the use of T2 data had been validated in a prior study as a marker for other gray matter abnormalities, notably vascular and iron deposition related changes, rather than just atrophy [1]. Therefore, the integration of these two volumetric modalities in the same experimental design and particularly in data analysis emerges as a relevant approach, because T2DM seems to affect both brain function and structure [8-11], leading to poorer performances in attention, memory, executive function, global cognitive status [12] and a higher risk of dementia [13]. In addition, this pathology is also related to brain vascular abnormalities, which include vascular hypertrophy in small arteries [14], lacunar infarcts [15] and microbleeds [16]. Furthermore, neglected vascular alterations may influence functional MRI data interpretation [17].

Thus this population is appropriate to demonstrate the basic outline of an inferential multivariate methods’ package developed directly within the SPM8 software framework. This study serves its purpose as an exercise to assess the potential to deploy a complete multivariate package, which may be used to complement the standard univariate analyses widely used in neuroimaging data analysis.

## 2 MATERIALS AND METHODS

### 2.1 Patient Selection

Thirty-four participants with T2DM and forty-two age- and gender- matched control participants were recruited. Controls were recruited from the general population of the University of Coimbra Hospital, University staff or from volunteers’ database of IBILI (Institute for Biomedical Imaging and Life Sciences), while T2DM patients were recruited at the Endocrinology Department of the Hospital. T2DM patients were diagnosed using standard criteria [18, 19]. Inclusion criteria for patient group: i) age between 40 and 75 years-old; ii) diabetes mellitus type 2 diagnosis at least one year prior to the commencement of the study. Inclusion criteria for the control group: i) age between 40 and 75 years-old; ii) diabetes mellitus type 2 diagnosis excluded based on levels of glycated hemoglobin (HbA1c); iii) diabetes mellitus type 2 diagnosis excluded based on fasting glucose.

An experienced neuroradiologist performed the neuroradiological assessment and examined the presence of white matter hyperintensities in T1- and T2-weighted MR images of all participants. Exclusion criteria for both groups were severe cerebrovascular disease (TIA or stroke), neurologic diseases unrelated to diabetes likely to affect cognitive functions, known history of psychiatric disease, and alcohol abuse. We finally included twenty-eight T2DM patients (mean age 58.1 ± 6.9 years) and twenty-six age- and gender- matched control (mean age 54.7 ± 7.3 years) participants.

### 2.2 Image Acquisition

The MR scans were acquired at the Portuguese Brain Imaging Network facilities in Coimbra, Portugal, on a 3T research scanner (Magnetom TIM Trio, Siemens) using a phased array 12-channel birdcage head coil (Siemens).

For each participant, a 3D anatomical MPRAGE (magnetization-prepared rapid gradient echo) scan was acquired using a standard T1-weighted gradient echo pulse sequence with TR = 2530 ms, TE = 3.42 ms, TI = 1100 ms, flip angle 7°, 176 single shot slices with voxel size 1x1x1 mm, and FOV 256 mm.

In addition, true 3D, high-resolution, T2-weighted images were also acquired. The turbo spin echo with variable flip-angle distribution (sampling perfection with application optimized contrasts using different flip angle evolution; SPACE) pulse sequence was used with the following scan parameters: TR/TE/NEX = 3200ms/450ms/2; matrix, 192x192x144 slices; voxel resolution 1.25x1.25x1.25mm. Parallel acquisition of independently-reconstructed images was allowed, using generalized, auto-calibrating, partially-parallel acquisitions to reduce specific absorption rate (SAR) and the scanning time.

### 2.3 Image Preprocessing

Both T1- and T2-weighted MR imaging scans were preprocessed using SPM8 (http://www.fil.ion.ucl.ac.uk/) , running on MATLAB R2012a^®^ (The Math-Works, Inc., Natick, MA), prior to subsequent univariate and multivariate statistical analyses. Firstly, all scans were previously reoriented, *i.e.* the image origin was set at the anterior commissure (AC) manually. In order to normalize, segment and modulate both T1 and T2 scans, the unified segmentation algorithm [20] was applied, which was the algorithm also used in the key reference paper listed above [1], where T2-VBM was validated. The images were spatially normalized to Montreal Neurological Institute (MNI) standard space by registering the MR images to the ICBM 152 template and then segmented into gray matter (GM), white matter (WM) and cerebrospinal fluid (CSF). The GM images were modulated to compensate for changes in GM volumes due to nonlinear registration. This accounts for local amount of expansion or contraction of brain structures, so that the total amount of GM/WM in the modulated images remains the same as it would be in the original images. For instance, if spatial normalization doubles the volume of a certain structure, then the correction will halve the intensity of the signal in that region. The total volume of tissue, in each structure, is corrected for individual brain size (tissue volume per unit volume of spatially normalized image) and can thus be compared. Additionally, the GM segments from both modalities were smoothed with an 8-mm smoothing kernel to ensure the normality of the data. The use of this algorithm in T2-weighted imaging has already been validated [1]. After the preprocessing steps, T1 and T2 images were in the same standardized space and had the same resolution: 2x2x2mm. Finally, these spatial normalized, segmented, modulated and smoothed images could be used for the voxel-wise statistical analyses using the univariate GLM [3], as well as for the multivariate approach described below.

### 2.4 Multivariate GLM

#### 2.4.1 Multivariate GLM Representation and Parameter Estimation

The multivariate GLM is a straightforward generalization of the univariate GLM, with assumptions that are thoroughly detailed in the work of McFarquhar *et al.* [7]. Compared to the univariate GLM, the multivariate version does not include only one vector of response variables (**Y**), rather this has a Y matrix where the number of the columns correspond to *p* dependent variables (DVs) to be used:

(1) $$Y_{n\times p}=X_{n\times k}\beta_{k\times p}+E_{n\times p.}$$

This relationship can be represented in matrix form as follows:

(2) $$\left[\begin{array}{ccc} y_{11} & \cdots & y_{1p}\\ \vdots & \ddots & \vdots \\ y_{\mathrm{n1}} & \cdots & y_{\mathrm{np}} \end{array}\right]=\left[\begin{array}{ccc} x_{11} & \cdots & x_{1k}\\ \vdots & \ddots & \vdots \\ x_{\mathrm{n1}} & \cdots & x_{\mathrm{nk}} \end{array}\right]\;\left[\begin{array}{ccc} \beta_{11} & \cdots & \beta_{1p}\\ \vdots & \ddots & \vdots \\ \beta_{\mathrm{k1}} & \cdots & \beta_{\mathrm{kp}} \end{array}\right]+\left[\begin{array}{ccc} \varepsilon_{11} & \cdots & \varepsilon_{1p}\\ \vdots & \ddots & \vdots \\ \varepsilon_{\mathrm{n1}} & \cdots & \varepsilon_{\mathrm{np}} \end{array}\right].$$

The number of columns in the **β** (regressors) and **E** (error) matrices match the number of *p* dependent variables. The number of columns of the design matrix **X** are the same as the number of *k* independent variables. The equations used to estimate the individual parameters and the residual errors ϵ are similar to the ones used in the univariate model *i.e.* $\hat{\beta }={\left(X^{T}X\right)}^{-1}X^{T}Y\;\mathrm{and}\;\hat{E}=Y-\hat{Y}\;(\mathrm{where}\;\hat{Y}=X\hat{\beta }\;\mathrm{are}\;\mathrm{fitted}\;\mathrm{values})$ , respectively [21].

The Box's M test [22] was used in a voxel-wise manner to test the null hypothesis that the observed covariance matrices of the dependent variables were equal across groups. We similarly applied Levene’s test for testing homogeneity of variance [23]. The voxels were only considered when the p-value of these tests were simultaneously larger than 0.05, which means that only the voxels with the covariance and variance matrices equal across the groups were accepted for the estimation step.

#### 2.4.2 Testing Multivariate GLM Hypothesis

As the **β** matrix has multiple columns of possible interest, it is possible to test linear hypotheses about these several columns. The general form of the hypothesis is then given by:

(3) $$H_{0}:A_{q\times k}\beta_{k\times p}{\;M}_{p\times l}-C=0,$$

where the *q* rows of **A** test hypotheses concerning the *k* independent variables, and the *l* columns of **M** test hypotheses concerning the *p* dependent variables. With these three matrices, a multivariate contrast matrix **C_q × l_** can be used to test several hypotheses regarding combinations of regressors [21].

As in the univariate model, the sum of squares regarding the hypothesis, i.e. the amount of variance associated with the contrast being tested, can be calculated using the following equations:

(4) $$B={\left(A\hat{B}M-C\right)}^{T}{\left[A{\left(X^{T}X\right)}^{-1}A^{T}\right]}^{-1}\left(A\hat{B}M-C\right),$$

(5) $$W=M^{T}\left({\hat{E}}^{T}\hat{E}\right)\;M.$$

These matrices are the sum of squares and cross products matrix between (**B**) and within (**W**) groups, respectively [21]. After the calculation of these matrices, the multivariate hypotheses may be tested in different ways, such as using the Hotelling-Lawley Trace, Roy’s Largest Root, Pillai’s Trace or Wilk’s Lambda [21]. The Roy’s Largest Root has been reported as a test that should be avoided because has much poorer Type I error given that it provides a lower-bound on the p-value. The others have been reported as similar [7]. Though any of these approaches could have been used, the Wilks’ Lambda was chosen because it presents the most balanced behavior, whereas the Hotelling-Lawley Trace is more liberal and the Pillai’s Trace is the most conservative (7):

(6) $$\wedge =\frac{\left|W\right|}{\left|W+B\right|}=\prod_{i}^{s}\frac{1}{1+\lambda_{i}}.$$

where the *λ_i_* values are given by the eigen values of **W^-1^ B**.

The *F*-ratio can be calculated using the approximation based on Wilk’s determinant criterion:

(7) $$F=\frac{1-\wedge^{1/t}}{\wedge^{1/t}}.\frac{\mathrm{rt}-2u}{\mathrm{lq}},$$

where *q* is the number of rows of **A** and *l* is the number of columns of **M**. The other parameters are given by:

(8) $$u=\frac{\mathrm{lq}-2}{4},$$

(9) $$r=n-k-\frac{l-q+1}{2},$$

(10) $$t=\left\{\begin{array}{cc} \sqrt{\frac{l^{2}q^{2}-4}{l^{2}+q^{2}-5}} & \mathrm{if}\;l^{2}+q^{2}-5\;>0\\ 1 & \mathrm{if}\;l^{2}+q^{2}-5\;\leq 0 \end{array}.\right.$$

where *n* is the sample size and *k* is the number of columns of the design matrix. The degrees of freedom of *F* are *lq* in the numerator and rt - 2*u* in the denominator. It also worth noticing that the *F*-value is exact when (l,q) ≤ 2. Finally, the *F*-value can be converted into an approximate p-value, allowing for the estimation of a significance map.

#### 2.4.3 Implementation of the Multivariate GLM

This particular setup of the multivariate GLM algorithm was implemented in MATLAB R2012a^®^ (The Math-Works, Inc., Natick, MA) and then, with the proper alterations in SPM8 framework, we could perform multivariate whole-brain analyses.

### 2.5 Alterations in SPM8 Interface

Currently, only univariate methods can be performed in SPM8. Thus, in order to perform multivariate analyses using SPM8 the following SPM8 functions were changed: *spm_cfg_con*, *spm_cfg_factorial_design, spm_conman*, *spm_contrasts*, *spm_design_factorial*, *spm_FcUtil, spm_getSPM*, *spm_list, spm_results_ui, spm_run_factorial_design* and *spm_spm.*

These alterations led to the creation of a new design menu Fig. (**1**), where several dependent variables (DVs) can be chosen: the user can choose the name and scans of each DV, as well as the number of levels and nuisance covariates.

Given the flexibility provided by the use of contrasts, their multivariate versions were also implemented. As such, a new contrast interface Fig. (**2**) was also created, where one partition for the M-contrast (contrast for multivariate procedures) can be found.

Altogether, these alterations made the insertion of the multivariate GLM algorithm possible (which could be used to calculate a Multivariate Analysis of Covariance – MANCOVA) and, to the best of our knowledge, this was the first time that a mass multivariate approach was implemented in SPM (see supplementary material for a description on how these alterations can be implemented). It is also worth noticing that these alterations do not interfere with the common univariate approaches in SPM.

### 2.6 Statistical Analyses

#### 2.6.1 Demographic and Clinical Data

Demographic and clinical data were analyzed using the Statistical Package for the Social Sciences software (SPSS version 19, IBM Corp. Released 2010. IBM SPSS Statistics for Windows, Version 19.0. Armonk, NY: IBM Corp). Continuous variables were tested for normality, separated by group, using the Kolmogorov-Smirnov test. Any rejection of the null hypothesis in any group was deemed an indication to use non-parametric tests for the analysis of that variable. Differences between the T2DM and control groups for non-normally distributed variables were tested using Mann-Whitney U test, while normally distributed variables were compared using Student’s t for two independent samples. For categorical variables, χ^2^ tests were applied. The results of these analyses can be seen in (Table **1**).

#### 2.6.2 Voxel-Wise Analyses

Gray matter alterations in T2DM patients were assessed by performing three distinct analyses using SPM: 1) an Analysis of Covariance (ANCOVA) with T1-weighted images; 2) an ANCOVA with T2-weighted images; 3) a MANCOVA, where T1- and T2-weighted images were used as DVs. In all the analyses, the positive contrast (controls > T2DM) was calculated and the total intracranial volume (TIV), known to be an important confound in VBM studies [24], was included as nuisance variable. The TIV was calculated in MATLAB using the T1 images and applying the automated method presented in [25]. After visually assessing the quality of the grey matter segments, a relative threshold mark of 0.20 was chosen as an adequate trade-off between structure preservation and grey matter boundary definition. We also computed the FDR (false discovery rate) corrected p-value threshold, but none of the analysis yielded significant voxels. Therefore, the statistical parametric maps were created using a more liberal uncorrected threshold (*p_uncorrected < 0.001_*) and an extent threshold *k = 10* voxels. The figures and tables showing the areas with significant GM alterations in T2DM patients were created using the xjview8 toolbox (http://www.alivelearn.net/xjview8/). Finally, for replication purposes, the aforementioned multivariate analysis was also performed using the MRM toolbox (http://www.click2go.umip.com/i/software/mrm.html) and the same p uncorrected threshold was used.

### 2.7 Ethical Standards and Patient Consent

We declare that the study was approved by the medical ethics committee of the University of Coimbra. Informed written consent was obtained from all participants. All clinical investigation was conducted according to the principles expressed in the Declaration of Helsinki of 1975 (and as revised in 1983).

## 3 RESULTS

### 3.1 ANCOVA with T1-Weighted Images

The voxel-wise statistical analysis reveals focal regions with less gray matter in T2DM patients, when compared with controls. Fig. (**3**) shows more pronounced bilateral alterations in basal ganglia, insula and thalami. The location and peak significance of the brain areas with significant less GM volume in T2DM patients are presented in (Table **2**).

### 3.2 ANCOVA with T2-Weighted Images

Fig. (**4**) illustrates abnormal grey matter in T2DM patients, when compared with controls. These findings suggest predominant bilateral alterations in basal ganglia and insular regions in T2DM patients. The location and significance of the peak voxels in regions with tissue differences are presented in (Table **3**).

Location and significance of peak voxels in regions with gray matter abnormalities in T2DM patients, using ANCOVA with T2-weighted images.

### 3.3 Overlap of T1 and T2-Weighted Images

Fig. (**5**) shows the maps with result of T1 and T2 overlaid in the same template brain. The red clusters correspond to the T1 analysis and blue clusters correspond to the T2 analysis. It is possible to identify the common (the purple clusters correspond to the overlapped areas) and disjointed affected areas between analyses.

### 3.4 Mancova

The result presented in Fig. (**6A**) was obtained by performing a MANCOVA in SPM, in which T1- and T2-weighted images were used as dependent variables simultaneously. Fig. (**6B**) shows the result of the MANCOVA analysis performed in the MRM toolbox. Both maps show a pattern of less grey matter volume and concomitant pathology such as inflammation or vascular in basal ganglia and thalami in T2DM patients when compared with controls. The location and significance of the peak voxels in these areas are presented in (Table **4**).

## DISCUSSION

This study presents a basic outline of an inferential multivariate package developed and implemented for the first time directly within the SPM8 software framework. As a proof of concept, we applied this mass multivariate approach on a group of T2DM patients, a pathology known to be related to brain structure and vasculature alterations.

The standard VBM approach only allows the detection of alterations in a specific imaging technique, *e.g.* using PET or MRI scans of a single modality. Given the different nature of the information obtained with each imaging modality, the use of standard univariate analyses alone is insufficient to give a full perspective of how these data interact and yield a more concise map of brain changes. Hypothetically, multivariate approaches can lead to a better understanding of imaging profiles of brain structure, activity and metabolism. As such, the multivariate GLM has been implemented in this work as an effort to facilitate multimodal analyses of imaging data. This natural extension of the commonly used massive univariate approach was used to integrate the information provided by multiple MRI sequences (T1 and T2 volumetric scans) of T2DM patients. The hypothesis was that the simultaneous analysis of distinct information obtained with each modality would allow for the better understanding of joint structural alterations and concomitant pathological changes, within the same statistical design.

Using the standard VBM approach, the similarities between the results obtained with T1 and T2 are striking. Although the former showed a spread pattern of less grey matter volume, the main alterations (basal ganglia and insula, bilaterally) are associated with alterations found in T2 analysis as well, in which these alterations seem to be more restrictive to those areas. Although the results were only significant at an uncorrected threshold, these findings are in line with previous studies [26, 27] and help further validating previous identified imaging biomarkers in diabetes, using univariate approaches.

**Fig. (3):**
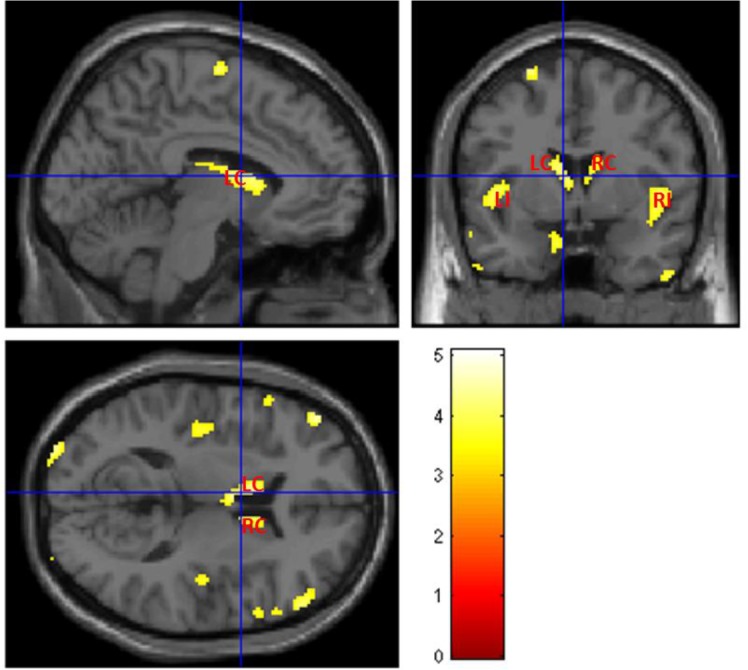
Areas with less grey matter volume in T2DM patients (p<0.001 single voxel, uncorrected, extent threshold k = 10) when compared with controls, using ANCOVA with T1-weighted images. The color bar indicates the range of t-values with white/yellow representing more significant differences (higher t-values), orange indicating less significant differences (middle range t-values) and red indicating non-significant differences (lower t-values). LC – Left Caudate; RC – Right Caudate; LI – Left Insula; RI – Right Insula.

The apparent overlap was confirmed in Fig. (**5**), where insula and the basal ganglia, bilaterally, are the key locations of tissue content changes in both modalities. This might reveal focal regions with less grey matter volume and concomitant pathology such as inflammation or vascular changes in these areas [27-29]. This was the starting point for the application of the multivariate inferential methods described above. Such methods have the potential for extracting additional information where the univariate methods see the same locations. Both MANCOVA approaches confirmed the pervasive alterations in the basal ganglia, notably in the caudate, and provided further information about the thalami Fig. (**6A** and **6B**) since this area only as appeared significantly altered in the T1 analysis and only on the left part of the brain (see Fig. **3** and Table **2**). The differences between the two maps are mainly due to two reasons: i) MRM analysis does not assume two thresholds that are calculated in our approach - relative and voxel extent thresholds; ii) the Box’s M and Levene’s test are applied before the model estimation in our approach, while in MRM these are only used in a post-estimation stage. To understand these differences, we decided to run again the analysis without checking the homogeneity of covariance and variance of each voxel before the estimation step (see supplementary material) and the result is very like the one obtained in MRM software. Therefore, we speculate that this could be the potential reason for the differences between the results of the MRM toolbox and our own work. Nonetheless, the most affected areas are highly comparable which is an indication of the validity of the results obtained with our approach.

Although the results were only significant at an uncorrected threshold, the pattern of changes is also in agreement with previous evidence of structural changes in T2DM. The involvement of the thalami has been previously reported in a meta-analysis study, and is a highly vascularized region known to be sensitive to vascular alterations [26]: the conjunction of both elements, less grey matter volume and vasopathies, justify this result of the multivariate analysis. As expected, the multivariate analysis requires larger statistical power, which may explain why a region such as the insula was no longer detected. We believe that this analysis emphasizes joint changes in both T1 and T2 images in other regions, as described above, and not in the insula [27, 30, 31]. It is sensible to speculate that both the basal ganglia and the thalami are greatly affected by both grey matter atrophy and vasopathies when compared to other brain regions, notably the insula. Further insights as to the contribution of each image modality to the results need to be addressed in the future and can be obtained by using *e.g.* descriptive linear discriminant analysis [7], as well as strategies to improve power [32].

Additionally, a drawback of the multimodal models is that the continuous covariates will probably deal with the confounding effects in the same way for both dependent variables, which can be challenging when those variables are unrelated. On the other hand, the multivariate GLM can handle dependent neuroimaging data (such as repeated measurement and multimodal imaging data at the group level) [7], which cannot be easily addressed with univariate approaches [6, 7]. Therefore, we recommend the use of both approaches to have a fuller picture of the underlying pathology. As our implementation of the multivariate GLM in SPM8 toolbox entailed the creation of identical menus already used for univariate GLM analyses, both approaches can be easily applied in a feedforward manner in the same framework.

As a proof of concept, this work still has a number of limitations. We have included in the implementation several key statistical assumptions underlying the multivariate model to ensure its correct application, such as variance-covariance homogeneity (using the Box’s M and Levene’s tests in a voxel wise manner), the correction for multiple comparisons (using the FDR correction) and the correlation amongst the dependent variables. Nevertheless, other post-estimation tools, such as the contribution of each image modality in the results and the ability to adequately apply resel-based family wise correction might be helpful for the interpretation of the results. Furthermore, the algorithm was adapted to these limited data, not taking into account difficulties that may arise from using multiple modalities (*e.g.* PET and fMRI), notably different scan space and resolution, which can be resolved by rescaling images of one modality to match another modality scan space. Additionally, the use of Box’s M and Levene’s tests before the model estimation might be a stringent approach as this can interfere with detecting true between group differences when using cluster based inference, because removing individual voxels from the images can break up clusters, rendering them insignificant (see supplementary material). Finally, although the proposed toolbox can correctly manage the univariate and multivariate analyses in the same framework, this is only possible by replacing some original functions of SPM. A standalone version of the multivariate toolbox should be prepared. Future work should validate the toolbox with a cohort where clear differences between T1 and T2 VBM can be seen a priori, thus facilitating the analyses and subsequent biological interpretation of results.

## CONCLUSION

This study presents multivariate methods that are a natural extension of the commonly used methods in standard massive univariate analyses of neuroimaging data. These were successfully implemented within the SPM8 software package, which is particularly relevant because both univariate and multivariate approaches can be applied in one of the most commonly used toolboxes in neuroimaging data analysis. Such multivariate approaches might be helpful tools to understand the complex mechanisms underlying brain alterations in some pathologies, which act across several dimensions (*e.g.* tissue structure and vascular) simultaneously rather than simply through brain atrophy. In the future, the inherent limitations of the algorithm should be addressed and it should be validated in other cohorts and expanded to accommodate other modalities, such as PET and fMRI.

## SUPPORTIVE/SUPPLEMENTARY MATERIAL

Tutorial for multivariate GLM analysis in SPM8 – software adaptation and procedure (word file).
The effect of Box's M and Levene’s tests in SPM maps.

## Figures and Tables

**Fig. (1):**
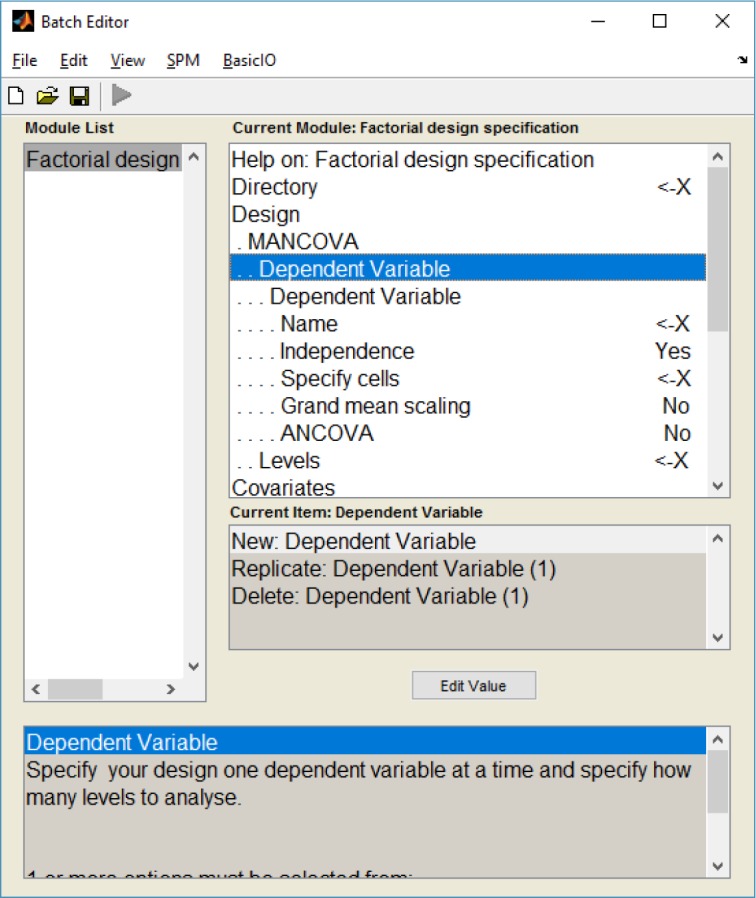
The new SPM8 design menu for the MANCOVA analysis.

**Fig. (2):**
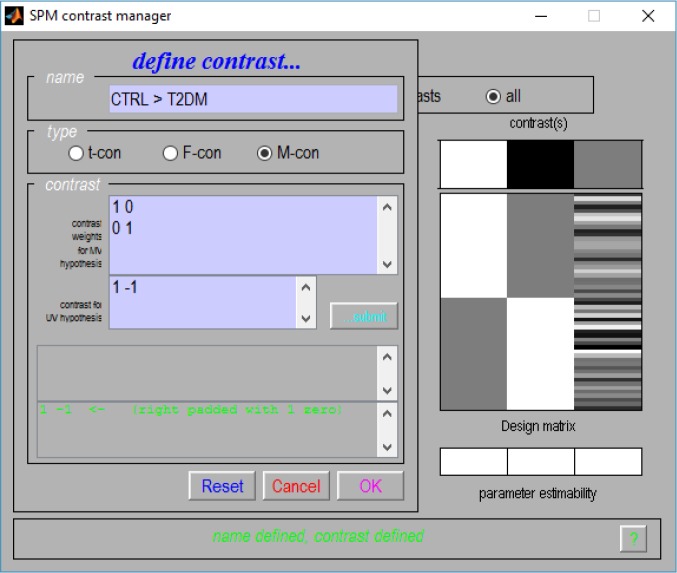
The new SPM8 contrast window for multivariate contrast definition.

**Fig. 4:**
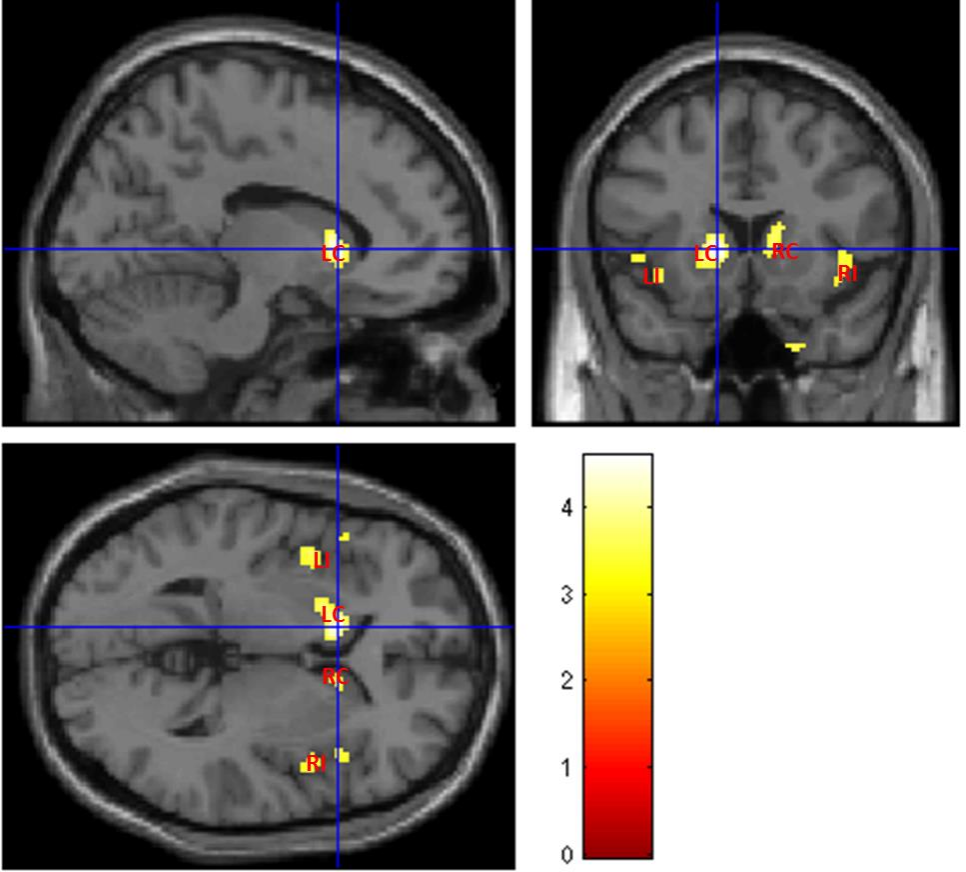
Areas with abnormal gray matter in T2DM patients (p<0.001, uncorrected, extent threshold k = 10) when compared with controls, using ANCOVA with T2-weighted images. The color bar indicates the range of t-values with white/yellow representing more significant differences (higher t-values), orange indicating less significant differences (middle range t-values) and red indicating non-significant differences (lower t-values). LC – Left Caudate; RC – Right Caudate; LI – Left Insula; RI – Right Insula.

**Fig. (5):**
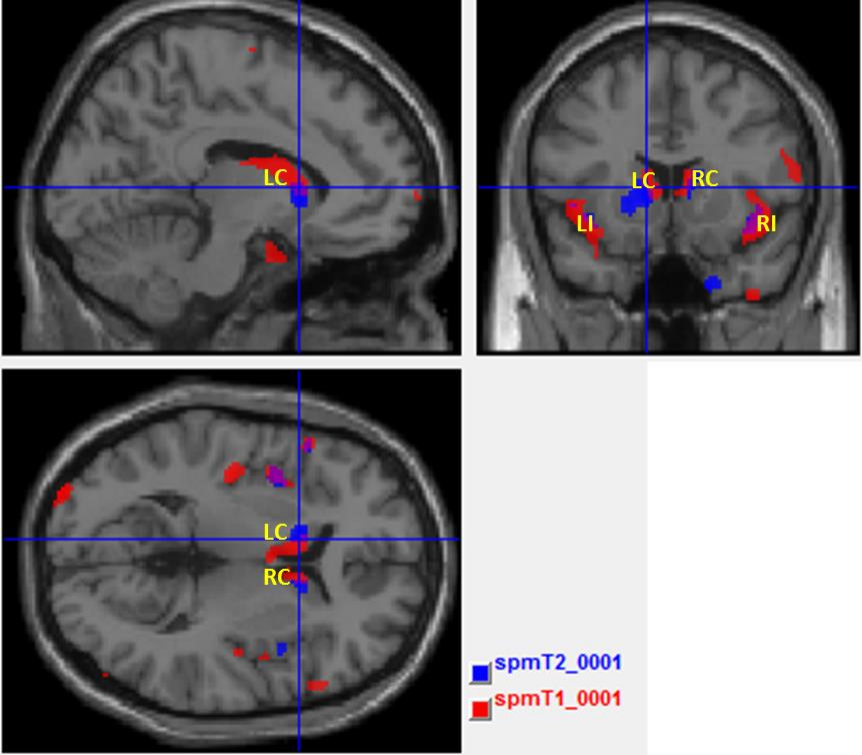
Overlapping of T1- (red clusters) and T2-weighted (blue clusters) univariate VBM analyses. The purple clusters correspond to the overlapped areas. LC – Left Caudate; RC – Right Caudate; LI – Left Insula; RI – Right Insula.

**Fig. (6):**
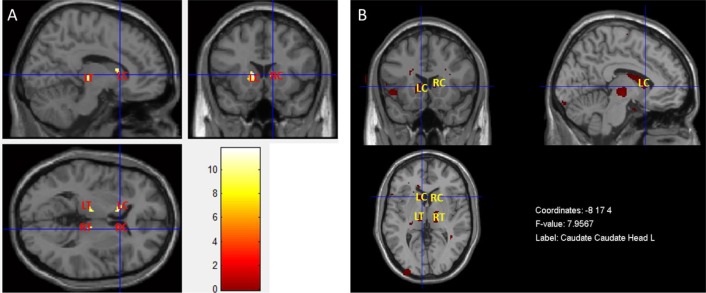
** (A)** Areas with less grey matter volume and concomitant pathology in T2DM patients (p<0.001, uncorrected and extent threshold k = 10) when compared with controls, using a MANCOVA design with T1 and T2 scans simultaneously, implemented in SPM (color bar represents the range of F-values calculated, in which red corresponds to lower F-values and yellow corresponds to greater F-values). **(B)** Areas with less grey matter volume and concomitant pathology in T2DM patients (p<0.001, uncorrected) when compared with controls, using the same data with a MANCOVA design in the MRM toolbox. LC – Left Caudate; RC – Right Caudate; LT – Left Thalami; RT – Right Thalami.

**Table 1:** Demographic and relevant clinical data from both groups.

**Variables**	**T2DM (n=28)**	**Controls (n=26)**	**Statistic; p-value**
Age (years, mean ± SD)	58.1 ± 6.9	54.7 ± 7.3	U = 256; >0.05
Gender (male: female)	17:11	13:13	*X^ 2^*_1_ = 0.627; >0.05
Body Mass Index (BMI) (kg/m2, mean ± SD)	29.16 ± 4.63	25.06 ± 2.60	***t* = -4.053; <0.001**
Blood glucose (mg/dL, mean ± SD)	164.29 ± 59.23	92.88 ± 11.05	***t* = -6.259; <0.001**
HbA1c (%, mean ± SD)	9.73 ± 3.01	5.38 ± 0.37	***t* = -7.457; <0.001**
Hypertension (HTM) (yes:no)	23:5	6:20	***X^ 2^*_1_= 18.92; <0.001**
TIV (dm^3^, mean ± SD)	1.51 ± 0.23	1.56 ± 0.20	*t* = 0.922; >0.05

**Table 2:** Location and significance of peak voxels in regions with less GM volume in T2DM patients, using ANCOVA with T1-weighted images.

**Number** **voxels**	**T-value**	**p (unc)**	**x, y, z (mm)**	**Areas**
548	4.9381	<0.00001	-48, 22, -4	Left Cerebrum, Frontal Lobe, Inferior Frontal Gyrus, Temporal Lobe, Inferior Frontal Gyrus, Left Insula
478	4.7138	<0.00001	42, 12, -12	Right Cerebrum, Frontal Lobe, Inferior Frontal Gyrus, Temporal Lobe, Right Insula, Middle Frontal Gyrus
320	4.2958	<0.0001	-4, 0, 10	Left Cerebrum, Left Caudate, Left Thalami
253	4.7791	<0.00001	-34, -98, 8	Left Cerebrum, Occipital Lobe, Middle Occipital Gyrus
167	3.857	<0.0001	8, 14, 10	Right Cerebrum, Right Caudate
165	4.0365	<0.0001	-54, -68, -36	Left Cerebrum, Cerebellum Posterior Lobe
148	4.6400	<0.0001	-22, -48, 68	Left Cerebrum, Parietal Lobe, Postcentral Gyrus, Brodmann area 5, 3, 2
148	5.0781	<0.00001	46, -14, 62	Right Cerebrum, Frontal Lobe, Parietal Lobe, Precentral Gyrus, Brodmann area 6, 4, 3

**Table 3:** Location and significance of peak voxels in regions with gray matter abnormalities in T2DM patients, using ANCOVA with T2-weighted images.

**Number** **voxels**	**T-value**	**p (unc)**	**x, y, z (mm)**	**Areas**
224	4.5757	<0.0001	-12, 16, 2	Left Cerebrum, Left Caudate, Lentiform Nucleus, Left Putamen
181	3.9073	<0.001	-48, 20, -2	Left Cerebrum, Left Insula, Frontal Lobe, Inferior Frontal Gyrus
123	3.8413	<0.001	30, -22, -30	Right Cerebrum, Limbic Lobe, Parahippocampa Gyrus, Right Parahippocampal
102	3.8719	<0.001	40, 12, -12	Right Cerebrum, Right Insula, Frontal Lobe, Inferior Frontal Gyrus, Temporal Lobe
57	3.7014	<0.001	14, 14, 12	Right Cerebrum, Right Caudate

**Table 4:** Location and significance of peak voxels of regions with less grey matter volume and concomitant pathology in T2DM patients, when using a MANCOVA with T1 and T2 data simultaneously in SPM.

**Number** **voxels**	**F-value**	**p (unc)**	**x, y, z (mm)**	**Areas**
54	11.58	<0.001	-16, 20, -2	Left Cerebrum, Left Caudate
39	11.31	<0.001	-6, -16, 0	Left Cerebrum, Left Thalami
31	11.75	<0.001	8, -14, -2	Right Cerebrum, Right Thalami
17	9.82	<0.001	14, 14, 14	Right Cerebrum, Right Caudate
