# Supplementary material for: Extending Inferential Group Analysis in Type 2 Diabetic Patients with Multivariate GLM Implemented in SPM8
Source: Open Neuroimag J. 2017 May 29;11:32–45. doi: 10.2174/1874440001711010032 (PMC5510560; doi:10.2174/1874440001711010032)
Supplement: Supplementary file 1 — Tutorial for multivariate GLM analysis in SPM8 – software adaptation and procedure (word file). The effect of Box's M and Levene’s tests in SPM maps. [file TONIJ-11-32_SD1.pdf]

Send Orders for Reprints to reprints@benthamscience.ae

BENTHAM OPEN

CrossMark

The Open Neuroimaging Journal

Supplementary Material

Content list available at: www.benthamopen.com/TONIJ/

DOI: 10.2174/1874440001711010032

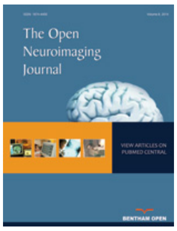

Extending Inferential Group Analysis in Type 2 Diabetic Patients with Multivariate GLM Implemented in SPM8

Fábio S. Ferreira[1,*], João M.S. Pereira[1,2,*], João V. Duarte[1,3] and Miguel Castelo-Branco[1,2,3,*]

[1]*Institute for Biomedical Imaging and Life Sciences (IBILI), Faculty of Medicine, University of Coimbra, Portugal*
[2]*Laboratory of Biostatistics and Medical Informatics, Faculty of Medicine, University of Coimbra, Portugal*
[3]*Institute for Nuclear Sciences Applied to Health (ICNAS), University of Coimbra, Portugal*

Received: November 22, 2016 Revised: December 07, 2016 Accepted: March 20, 2017

TUTORIAL FOR MULTIVARIATE GLM ANALYSIS IN SPM8 – SOFTWARE ADAPTATION AND PROCEDURE

In this tutorial the necessary steps to perform a multivariate analysis in any computer with a Matlab version compatible with SPM8 are explained, assuming that the images are already spatially preprocessed.

1. Go to http://www.fil.ion.ucl.ac.uk/spm/software/spm8/ and download the latest version of SPM8 software.
2. After the installation process, go to http://www.uc.pt/en/fmuc/ibili/Archives/app/MultivariateGLM. The “MultivariateGLM” zip file will be automatically downloaded.
3. Extract the new functions *(spm_cfg_con, spm_cfg_factorial_design, spm_run_factorial_design, spm_conman, spm_contrasts, spm_design_factorial, spm_FcUtil, spm_getSPM, spm_leveneTest, spm_list, spm_MBoxtest, spm_results_ui, spm_spm)* from “MultivariateGLM” zip file and insert them in the spm8 folder. The first three functions must be pasted inside of the *config* folder. **Note:** we advise you to save the old functions before you overwrite them with the new ones. This will allow you to reset the toolbox anytime you want.
4. Start the Matlab program and write ‘spm’ in the command window. The following window will appear:

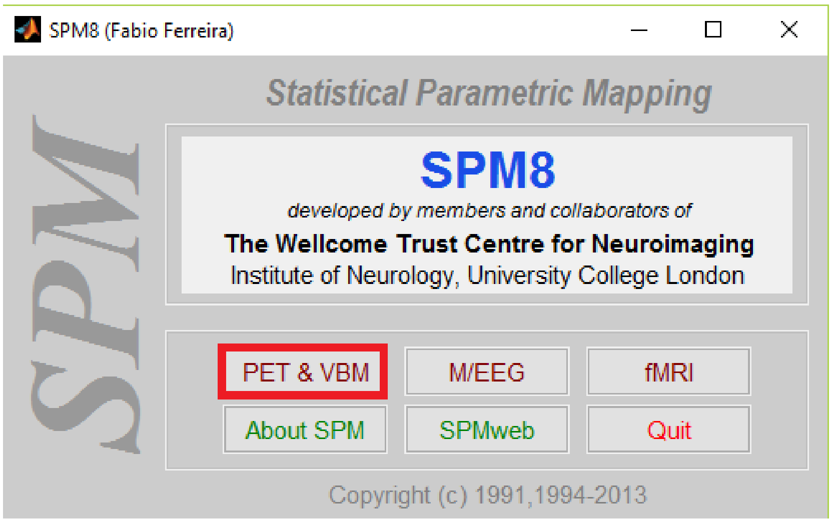

5. Choose the ‘PET & VBM’ button and the following window will appear:

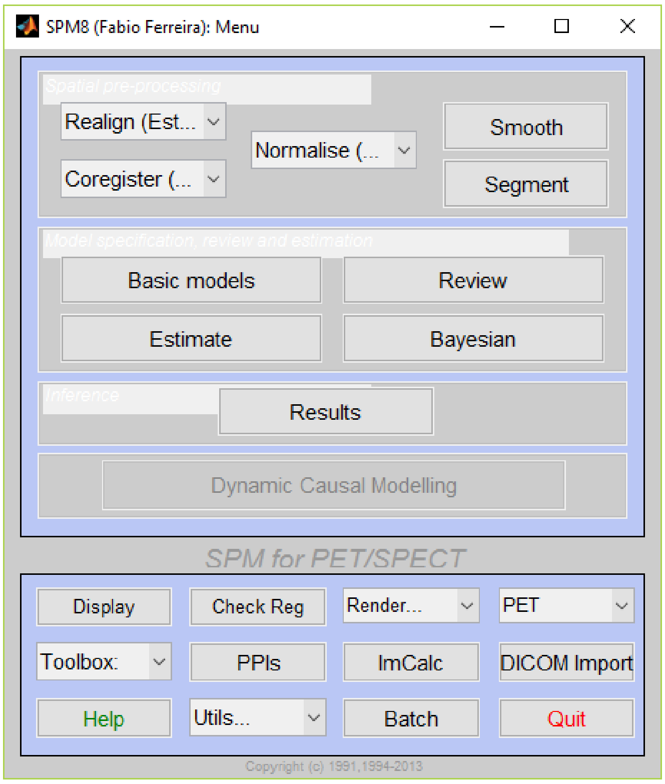

6. Click on the 'Basic models' button and the design menu window will appear:

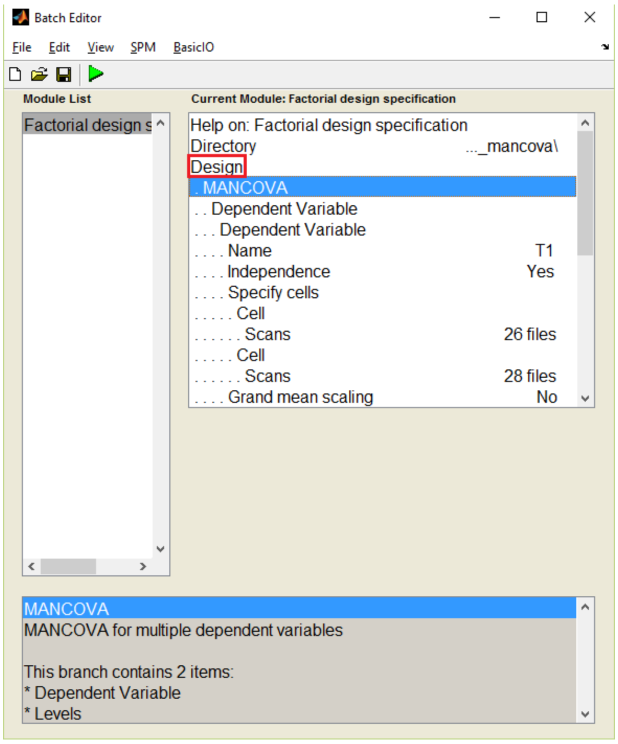

Here it is possible to choose the directory where the SPM.mat file, with specified design matrix, will be written, as well as the intended design. In this case, choose the 'MANCOVA' design.

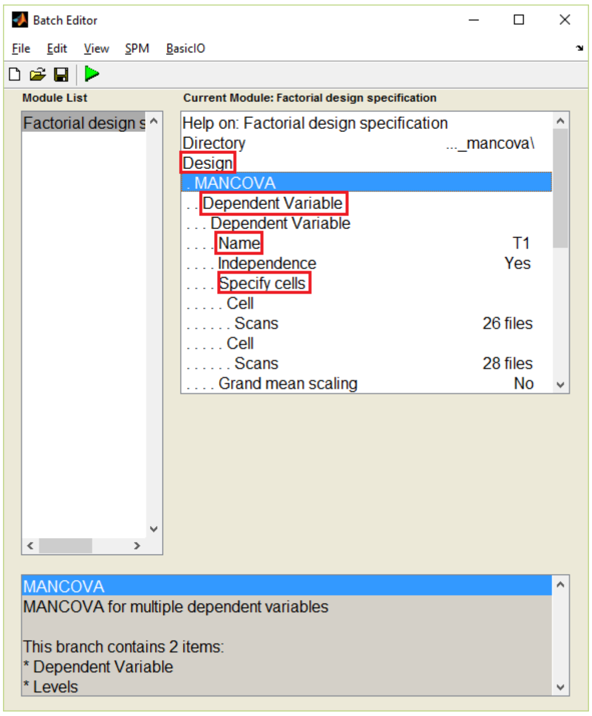

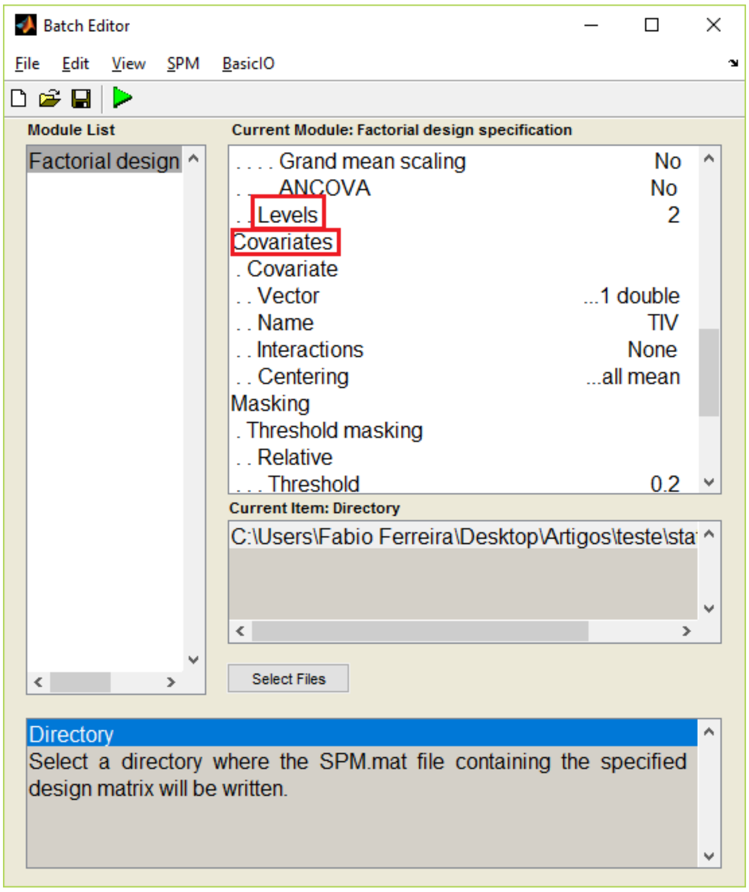

In this design, multiple dependent variables (DVs) can be chosen: it is possible to choose the name of the DV, associate with each DV the scans to analyze, as well as the number of levels and the nuisance covariates, among

other options.

7. After the insertion of all design specifications, click on the 'run' button (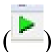) to create the SPM.mat file.
8. Go to the main window (PET & VBM window), press the 'Estimate' button and select the SPM.mat file created previously (this lead to the estimation of SPM.mat file).
9. Then click on 'Results' button and choose the estimated SPM.mat file. The contrast manager window will become visible:

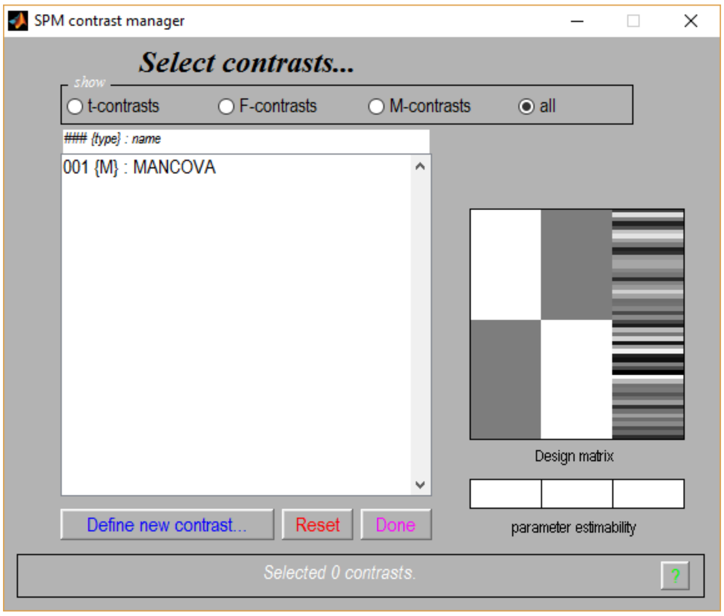

10. Select the 'M-contrasts' button and click on 'Define new contrast...' button:

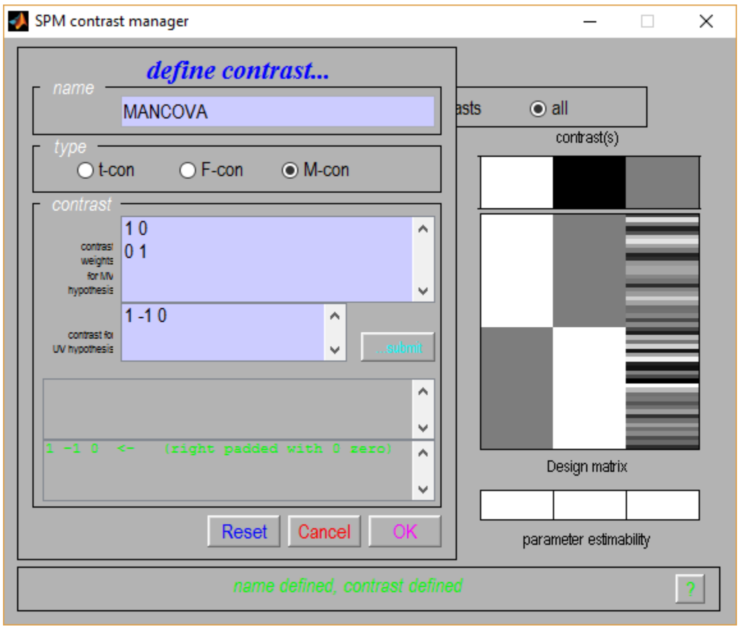

11. Here, the multivariate contrast can be defined. In the upper rectangle the contrast name can be inserted. In the second rectangle, the contrast weights to test the dependent variables can be inserted; the third rectangle requires the input of the contrast weights to test the independent variables. After this, press the 'submit' button and finally 'OK' button. In order to give the users a better understanding of the contrasts weights, we have made an example for you: we have two dependent variables (gray matter T1 and T2 images), two independent variables

(controls and diabetic patients) and one nuisance variable (total intracranial volume). The contrast $\begin{bmatrix} 1 & 0 \\ 0 & 1 \end{bmatrix}$ in the second rectangle will allow the model to calculate the joint effects of the two dependent variables (in this case, modulated gray matter a posteriori probability of T1 and T2 images) and the contrast [1 - 1 0] in the third rectangle will allow the model to compare the differences in gray matter volume between controls and diabetic patients, using the total intracranial volume as a covariate.

12. The contrast manager window will appear again. Select the created contrast and press 'Done'. The following window will appear:

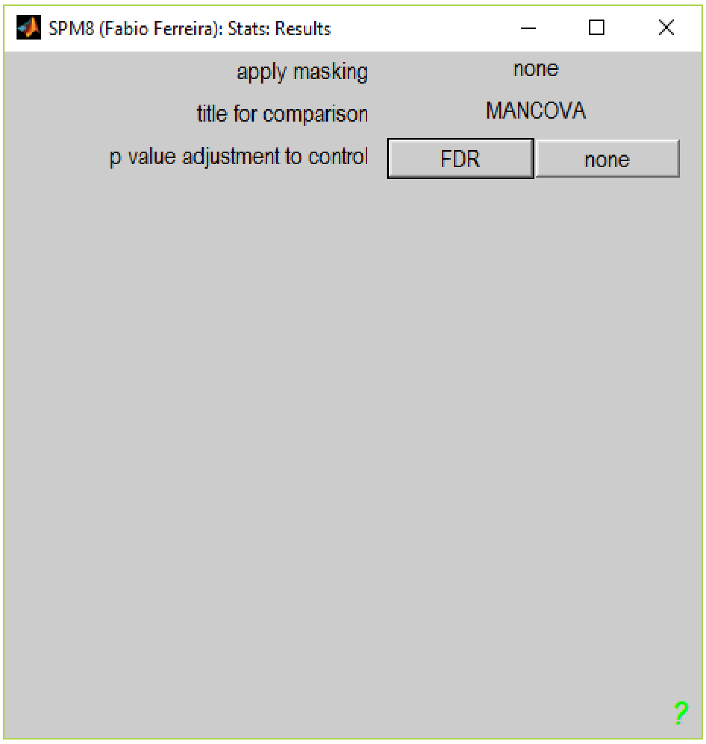

13. Insert the intended parameters for the analysis and press Enter. Wait until the end of the calculation and then you can choose an extent threshold (cluster size control):

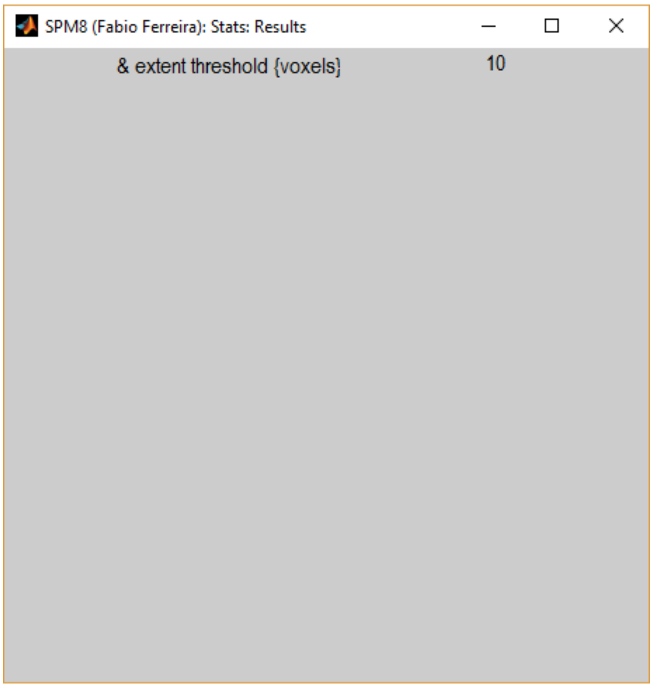

15. After this a result such as the following one should appear:

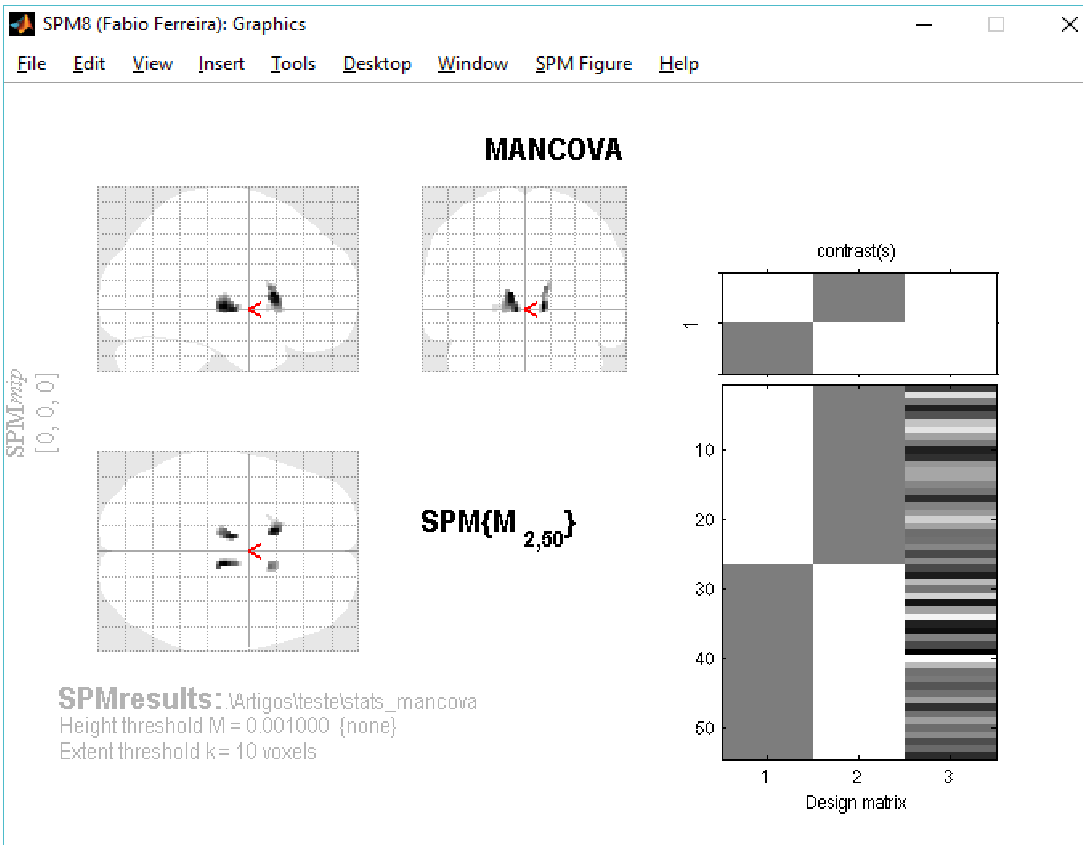

Finally, in the results window, the previous result can be overlaid with a template. For this, click ‘overlays...’, select the tag ‘sections’ and then choose a template in the spm8 folder:

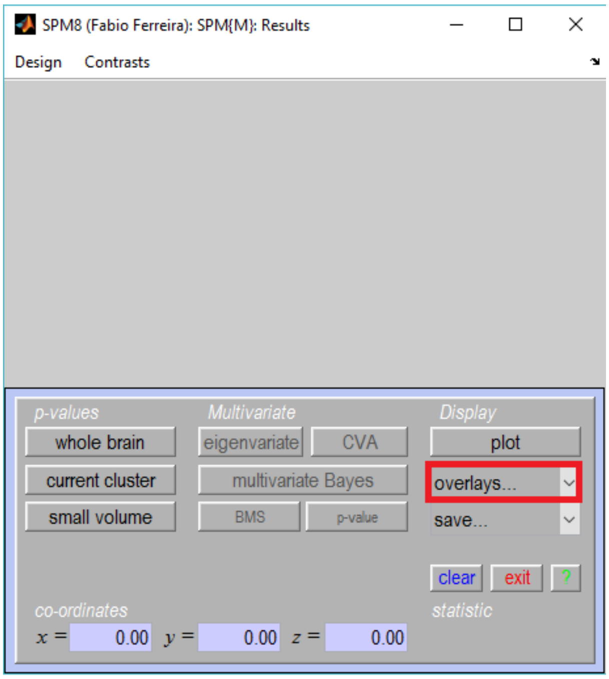

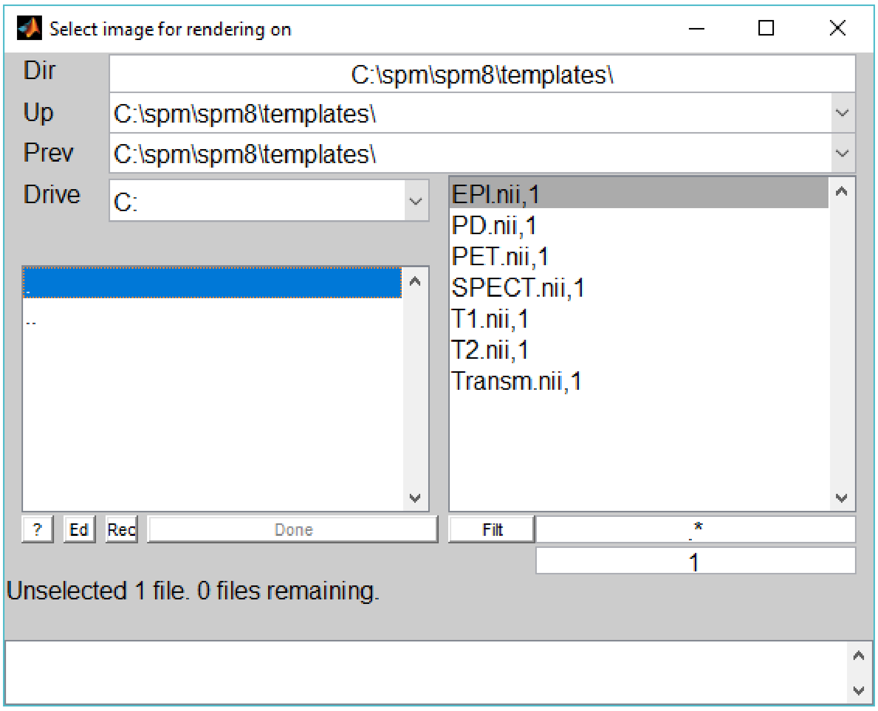

16. After all these steps, a similar result can be obtained:

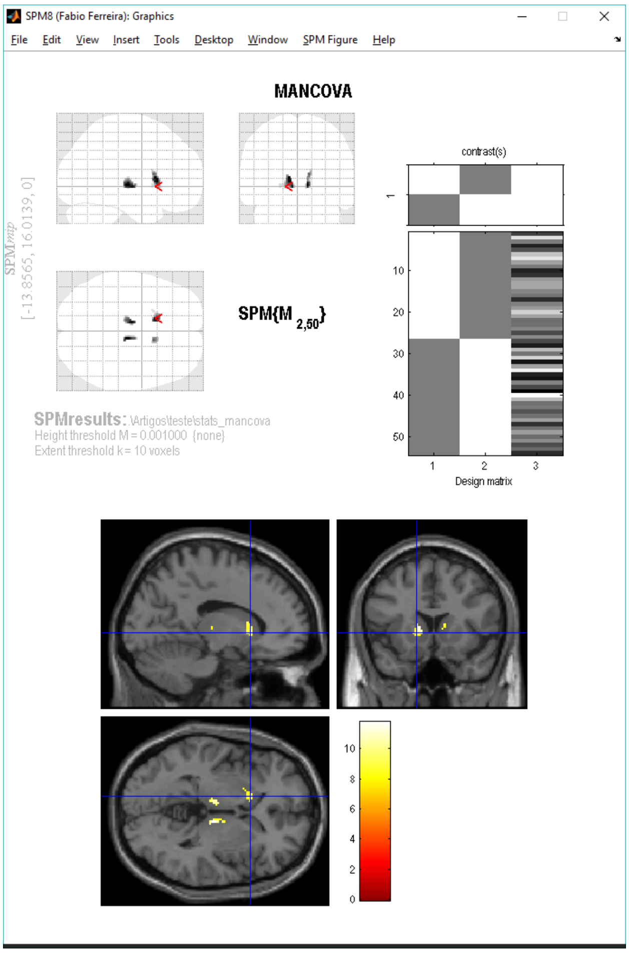

THE EFFECT OF BOX'S M AND LEVENE'S TESTS IN SPM MAPS

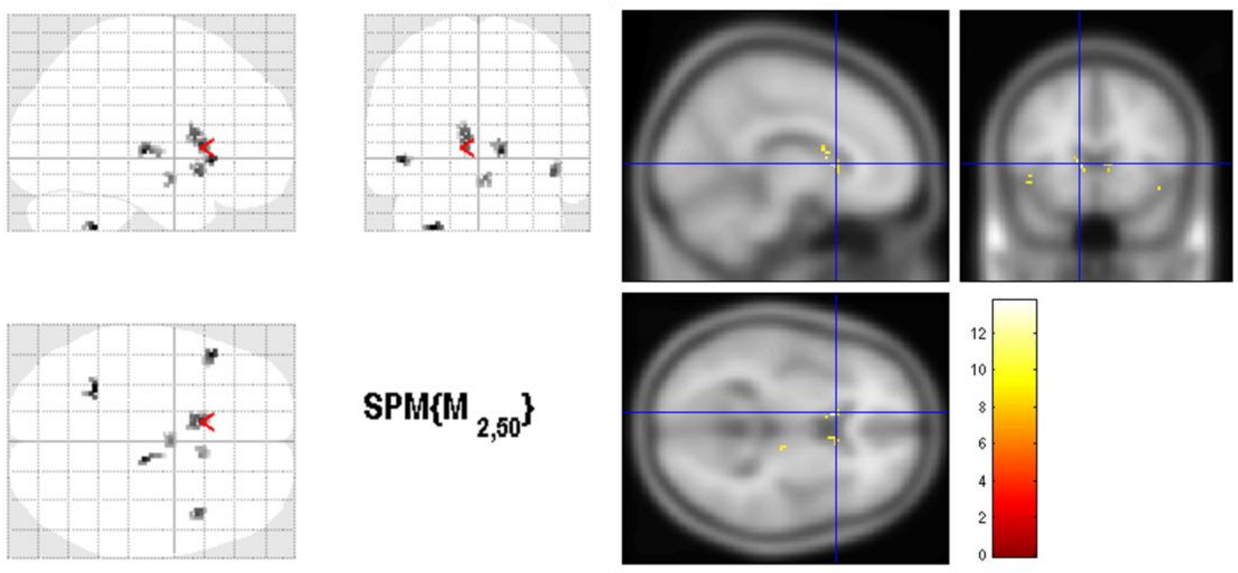

Fig. (1). Voxels excluded by applying Box's M and Levene's tests before the estimation step.

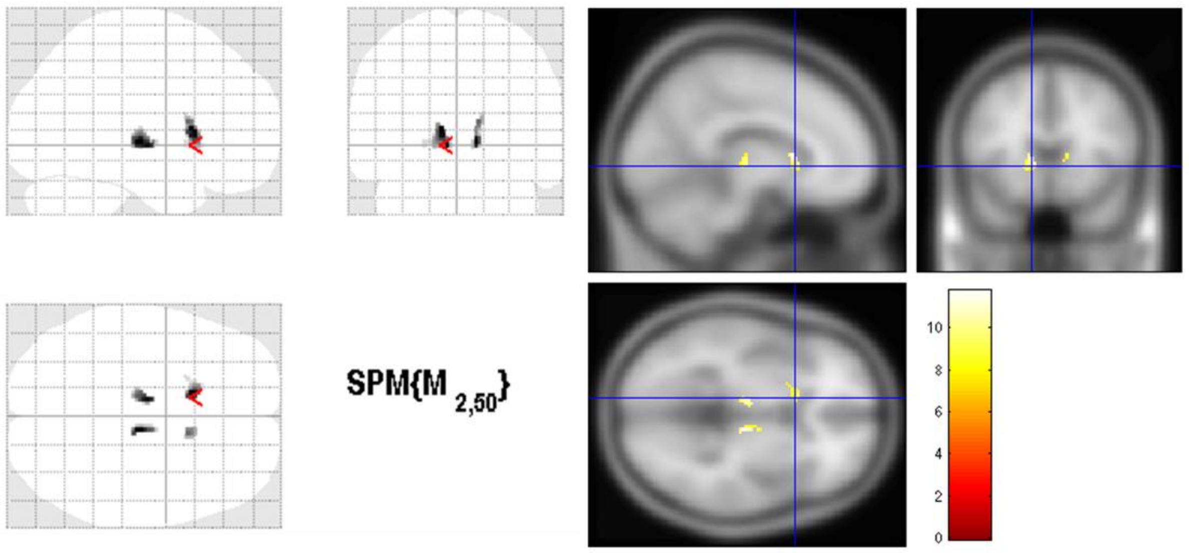

Fig. (2). Voxels included by applying Box's M and Levene's tests before the estimation step.

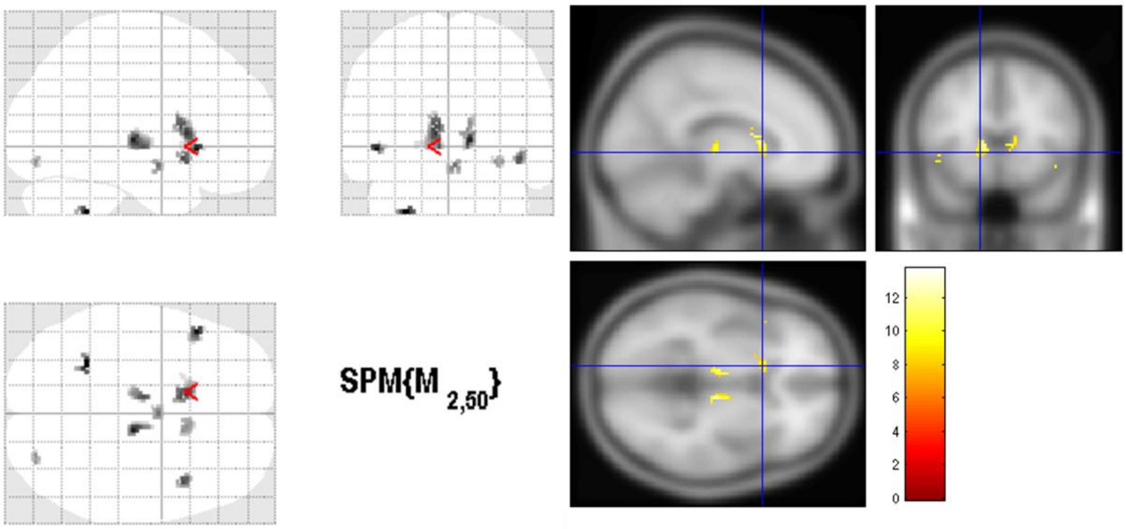

Fig. (3). Areas with less grey matter volume and concomitant pathology in T2DM patients (p<0.001, uncorrected, extent threshold k = 10) when Box's M and Levene's tests were not used.

© 2017 Ferreira *et al.*

This is an open access article distributed under the terms of the Creative Commons Attribution 4.0 International Public License (CC-BY 4.0), a copy of which is available at: https://creativecommons.org/licenses/by/4.0/legalcode. This license permits unrestricted use, distribution, and reproduction in any medium, provided the original author and source are credited.
